# Supplementary material for: The Short-Term Impact of Botulinum Neurotoxin-A on Muscle Morphology and Gait in Children with Spastic Cerebral Palsy
Source: Toxins (Basel). 2022 Sep 29;14(10):676. doi: 10.3390/toxins14100676 (PMC9607504; doi:10.3390/toxins14100676)
Supplement: Supplementary file 1 [file toxins-14-00676-s001.zip › toxins-1910827-supplementary.pdf]

toxins

MDPI

Table S1. Muscle morphology parameters of both the intervention and control group.

Part I – Primary outcomes											
	Intervention group					Control group					
parameter – treated muscles	pre	post	Median difference post-pre	Median within group, percent change	p-value, within group	pre	post	Difference post-pre	Median within group, percent change	p-value, within group	p-value between groups
MG – muscle volume (ml/kg*m)	1.40 (1.03 – 1.60)	1.31 (0.99 – 1.54)	-0.08 (-0.16 – 0.04)	-5.15	0.018	1.23 (1.04 – 1.65)	1.29 (1.02 – 1.55)	0.00 (-0.09 – 0.06)	-0.25	0.841	0.118
MG – echogenicity intensity (AU)	166.75 (158.78 – 180.19)	174.17 (159.08 – 186.04)	2.74 (-5.63 – 11.11)	1.46	0.407	165.28 (152.60 – 178.02)	165.66 (157.43 – 179.37)	-0.08 (-3.77 – 9.33)	-0.50	0.455	0.814
MG Growth rate – absolute (ml/month)	0.00 (-1.34 – 0.97)					0.17 (-0.23 – 0.87)					0.346
ST – muscle volume (ml/kg*m)	0.68 (0.57 – 0.73)	0.57 (0.47 – 0.69)	-0.09 (-0.16 – 0.01)	-16.18	0.030	0.68 (0.63 – 0.78)	0.70 (0.58 – 0.75)	-0.01 (-0.07 – 0.04)	-1.21	0.445	0.069
ST – echogenicity intensity (AU)	151.45 (142.13 – 168.47)	165.42 (149.90 – 172.55)	6.58 (-8.27 – 16.61)	4.92	0.145	149.90 (145.00 – 161.30)	158.11 (149.20 – 166.87)	5.20 (-6.90 – 11.90)	3.54	0.227	0.728
ST Growth rate – absolute (ml/month)	-0.49 (-1.45 – 0.58)					0.02 (-0.39 – 0.48)					0.226
Part II – Secondary outcomes											
	Intervention group					Control group					

parameter – treated muscles	pre	post	Median difference post-pre	Median within group, percent change	p-value, within group	pre	post	Difference post-pre	Median within group, percent change	p-value, within group	p-value between groups
MG – MTU (%)	45.54 (44.06 – 45.91)	46.37 (44.61 – 46.96)	0.88 (-0.22 – 1.59)	2.05	0.011	46.23 (45.21 – 47.39)	46.35 (45.40 – 46.91)	-0.35 (-0.86 – 0.52)	-0.74	0.286	0.008
MG – muscle belly length (%)	22.86 (21.49 – 25.17)	23.23 (21.69 – 25.66)	0.44 (0.03 – 1.25)	2.14	0.009	24.04 (22.44 – 26.12)	24.16 (22.47 – 25.83)	-0.13 (-0.92 – 0.71)	-0.50	0.811	0.033
MG – tendon length (%)	21.57 (19.60 – 23.00)	21.79 (19.62 – 24.25)	-0.03 (-0.88 – 1.17)	-0.15	0.568	22.04 (20.93 – 23.59)	21.91 (20.72 – 23.46)	-0.26 (-0.55 – 0.24)	-1.10	0.157	0.2374
MG Growth rate – normalized (ml/kg/month)	-0.02 (-0.07 – 0.03)					0.01 (-0.04 – 0.02)					0.248
ST – muscle belly length, total (%)	34.26 (31.07 – 35.97)	34.63 (31.07 – 35.97)	0.15 (-0.87 – 0.97)	0.43	0.809	33.98 (31.66 – 35.55)	34.33 (31.64 – 36.04)	-0.10 (-1.06 – 1.82)	-0.28	0.865	0.684
ST – muscle belly length, proximal (%)	21.71 (20.16 – 24.04)	22.58 (20.77 – 23.65)	0.76 (-2.19 – 2.46)	3.13	0.778	22.03 (19.71 – 23.59)	21.26 (19.81 – 24.35)	0.14 (-0.86 – 1.27)	0.58	0.820	0.639
ST – muscle belly length, distal (%)	22.74 (21.61 – 24.79)	22.78 (21.34 – 24.54)	-0.32 (-0.90 – 1.47)	4.92	0.681	23.17 (21.78 – 26.21)	23.84 (21.48 – 26.28)	-0.22 (-0.74 – 0.83)	-0.91	0.687	0.675
ST Growth rate – normalized (ml/kg/month)	-0.02 (-0.08 – 0.01)					-0.00 (-0.03 – 0.02)					0.138

Kg = kilograms, m = meter, MG = medial gastrocnemius, ml = milliliters, MTU = muscle tendon unit length, ST = semitendinosus

Table S2. Results of the gait kinematics, gait profile and gait variable score and the spatial-temporal parameters, intervention group.

**Part I – Primary outcomes**					
**Gait**	**Intervention group**				
**Kinematic parameters - Ankle**	**pre**	**post**	**Median difference post-pre**	**p-value, treatment induced/within group**	**SEM** (1)
**ankle ROM in sagittal plane (total GC)** (degrees)	25.85 (19.83 – 34.55)	24.18 (21.51 – 29.13)	-1.74 (-9.04 – 3.07)	0.143	3.7
**ankle angle in sagittal plane at IC** (degrees)	-3.83 (-11.13 – 2.14)	-2.25 (-5.25 – 4.94)	3.56 (0.47 – 6.36)	0.002	4.4
**max ankle angle (max DF) in sagittal plane during stance phase** (degrees)	9.47 (0.70 – 14.52)	12.89 (7.37 – 17.76)	3.99 (-0.32 – 9.99)	0.004	4.7
**max ankle angle (max DF) in sagittal plane during swing phase** (degrees)	-1.71 (-9.63 – 3.12)	2.53 (-3.66 – 7.03)	5.34 (1.16 – 9.70)	<0.001	4.7
**Kinematic parameters - Knee**	**pre**	**post**	**Median difference post-pre**	**p-value, treatment induced/within group**	**SEM** (1)
**knee ROM in sagittal plane** (degrees)	53.50 (45.13 – 62.03)	54.84 (46.85 – 60.12)	1.94 (-3.79 – 8.84)	0.158	5.2
**knee angle in sagittal plane at IC** (degrees)	26.45 (20.93 – 35.43)	25.53 (17.26 – 30.53)	-1.69 (-9.19 – 1.84)	0.048	3.5
**min knee angle in sagittal plane during stance** (degrees)	5.06 (0.79 – 14.05)	6.74 (-1.22 – 10.09)	-2.44 (-7.61 – 2.23)	0.065	3.9
**Gait profile score**	**pre**	**post**	**Median difference post-pre**	**p-value, treatment induced/within group**	**MCID** (2)
**Gait profile score for angles** (degrees)	9.80 (7.41 – 11.33)	8.30 (6.71 – 9.41)	-1.76 (-2.51 - -0.15)	0.005	1.6

Spatial-temporal parameters	pre	post	Median difference post-pre	p-value, treatment induced/within group	SEM (1)
**Cadence** (number of steps/second)	2.25 (1.95 – 2.43)	2.19 (1.91 – 2.35)	-0.05 (-0.28 – 0.10)	0.156	11.7
**Walking velocity** (meter/second)	0.89 (0.67 – 1.10)	0.85 (0.73 – 1.05)	0.01 (-0.10 – 0.10)	0.911	0.1
**Stride length** (meters)	0.81 (0.66 – 0.96)	0.79 (0.69 – 1.04)	0.02 (-0.03 – 0.08)	0.108	0.1
**Part II – Secondary outcomes**					
**Gait**	**Intervention group**				
**Kinematic parameters**	**pre**	**post**	**Median difference post-pre**	**p-value, treatment induced/within group**	**SEM** (1)
**ankle ROM in sagittal plane during stance phase** (degrees)	16.85 (12.89 – 21.38)	15.92 (14.03 – 20.10)	0.31 (-4.38 – 3.74)	0.737	3.7
**knee ROM in sagittal plane during stance** (degrees)	21.69 (17.75 – 29.74)	26.86 (22.55 – 31.20)	3.68 (-0.43 – 6.91)	0.028	5.2

DF = dorsiflexion, GC = gait cycle, IC = initial contact, MCID = minimally clinically important difference, ROM = range of motion, SEM = standard error of measurement

Table S3. Results of the clinical examination in the intervention group.

**Clinical examination – treated muscles**	**Intervention group**			
**parameter**	**pre**	**post**	**Median difference post-pre**	**p-value, treatment induced/within group**
**ROM plantar flexors, knee extended**	5.0 (0.0 – 10.0)	5.0 (0.0 – 10.0)	0.0 (0.0 – 5.0)	0.302
**MAS plantar flexors, knee extended**	2.0 (2.0 – 3.0)	2.0 (1.5 – 2.0)	0.0 (-1.0 – 0.0)	n.a.
**MTS plantar flexors, knee extended**	-15.0 (-21.3 - -13.8)	-10.0 (-15.0 - -5.0)	5.0 (0.0 – 15.0)	0.027
**MAS plantar flexors, knee 90 degrees**	2.0 (1.5 – 2.0)	1.5 (1.5 – 1.5)	0.0 (0.0 – 0.5)	n.a.
**MTS plantar flexors, knee 90 degrees**	-10.0 (-12.5 - - 5.0)	-5.0 (-10.0 – 0.0)	0.0 (0.0 – 8.8)	0.085
**Popliteal angle, unilateral**	-60.0 (-65.0 - -45.0)	-55.0 (-65.0 - -48.8)	0.0 (-5.0 – 1.3)	0.564
**MAS knee flexors**	1.5 (1.0 – 1.75)	1.5 (1.0 – 1.5)	0.0 (-0.5 – 0.0)	n.a.
**MTS knee flexors**	-82.50 (-90 - -70)	-77.50 (-80.00 - -70.00)	5.0 (0.0 – 10.0)	0.048

MAS = modified Ashworth score, MTS = modified Tardieu score, n.a. = not applicable, ROM = range of motion

Table S4. Results of the instrumented spasticity assessment of a subgroup (*n*=14) in the intervention group.

**Part I – Primary outcomes**					
**Instrumented spasticity assessment – treated muscles**	**Intervention group (*n*=14)**				
**parameter**	**pre**	**post**	**Median difference post-pre**	**p-value, treatment induced/ within group**	**SEM** intra rater
**Medial gastrocnemius, EMG – absolute high rms-EMG** (µV)	11.52 (4.87 – 22.07)	4.91 (3.67 – 9.45)	-4.73 (-15.74 – 1.31)	0.019	unknown
**Medial hamstrings, EMG – absolute high rms-EMG** (µV)	12.07 (5.77 – 16.04)	7.98 (3.68 – 10.58)	-2.82 (7.64 – 0.21)	0.041	unknown
**Part II – Secondary outcomes**					
**Instrumented spasticity assessment – treated muscles**	**Intervention group (*n*=14)**				
**parameter**	**pre**	**post**	**Median difference post-pre**	**p-value, treatment induced/ within group**	**SEM**(3,4) intra rater
**Medial gastrocnemius, EMG – change rms-EMG** (µV)	5.10 (2.17 – 12.67)	3.52 (3.07 – 6.59)	-2.64 (-8.06 – 2.48)	0.101	2.0
**Medial hamstrings, EMG – change rms-EMG** (µV)	6.94 (4.47 – 13.19)	4.44 (1.44 – 9.39)	-3.13 (-5.78 – 0.87)	0.060	1.0

EMG = electromyography, rms = root mean square, SEM = standard error of measurement, µV = microvolts

1. Klejman S, Andrysek J, Dupuis A, Wright V. Test-Retest Reliability of Discrete Gait Parameters in Children With Cerebral Palsy. Arch Phys Med Rehabil [Internet]. 2010 May;91(5):781–7. Available from: http://dx.doi.org/10.1016/j.apmr.2010.01.016
2. Baker R, McGinley JL, Schwartz M, Thomason P, Rodda J, Graham HK. The minimal clinically important difference for the Gait Profile Score. Gait Posture [Internet]. 2012

Apr;35(4):612–5. Available from: http://www.ncbi.nlm.nih.gov/pubmed/22225850

3. Schless S, Desloovere K, Aertbeliën E, Molenaers G, Huenaerts C, Bar-On L. The Intra- and Inter-Rater Reliability of an Instrumented Spasticity Assessment in Children with Cerebral Palsy. PLoS One [Internet]. 2015;10(7):e0131011. Available from: http://www.ncbi.nlm.nih.gov/pubmed/26134673

4. Bar-On L, Aertbeliën E, Molenaers G, Van Campenhout A, Vandendoorent B, Nieuwenhuys A, et al. Instrumented assessment of the effect of Botulinum Toxin-A in the medial hamstrings in children with cerebral palsy. Gait Posture [Internet]. 2014 Jan;39(1):17–22. Available from: http://www.ncbi.nlm.nih.gov/pubmed/23791154
